# Characteristics of cardio-Cerebrovascular modulation in patients with generalized anxiety disorder: an observational study

**DOI:** 10.1186/s12888-017-1428-6

**Published:** 2017-07-18

**Authors:** Zhen-Ni Guo, Liangshu Feng, Xiuli Yan, Le Yang, Shuo Huang, Yingqi Xing, Yi Yang

**Affiliations:** 1 Clinical Trail and Research Center for Stroke, Department of Neurology, the First Hospital of Jilin University, Chang Chun, China; 2 Department of Neurology, the First Hospital of Jilin University, Xinmin Street 71#, Changchun, 130021 China; 3 Department of transcranial doppler, Liaocheng People Hospital, Liaocheng, Shandong China; 4 Center for Neurovascular ultrasound, the First Hospital of Jilin University, Chang Chun, China

## Abstract

**Background:**

Generalized anxiety disorder (GAD) has been shown in previous studies to display abnormal cerebral blood flow velocity (CBFV); however, the characteristics of cardio-cerebrovascular modulation are unknown. We aimed to analyze cardio-cerebrovascular modulation using parameters from a supine-to-standing test.

**Methods:**

There are 2 parts to this study; in Part 1, 125 participants with Hamilton Anxiety scale scores ≥14 were enrolled, and 33 age- and sex-matched medically and psychiatrically healthy volunteers were recruited as control participants. Patients were divided by score into mild, moderate, and severe anxiety groups. The cardio-cerebrovascular modulation using the parameters of dynamic changes of CBFV and heart rate in response to an orthostatic challenge were investigated. In Part 2, we followed up the severe GAD patients for 6 months and repeated the supine-to-standing test, and severe GAD patients were divided into recovery and non-recovery groups.

**Results:**

In part 1, the GAD group displayed more marked CBFV and heart rate changes than the healthy group, but there was no difference in the CBFV and heart rate changes from the supine to upright position in mild, moderate, and severe anxiety groups. In part 2, The recovery group demonstrated significant improvement in changes in the CBFV and heart rate values from the supine to the upright position after treatment compared with before treatment. In the non-recovery group, the CBFV and heart rate changes were significantly higher than the healthy group regardless of treatment.

**Conclusions:**

Cardio-cerebrovascular modulation is compromised in patients with GAD, however, this impairment can be restored to normal after the disappearance of anxiety.

**Electronic supplementary material:**

The online version of this article (doi:10.1186/s12888-017-1428-6) contains supplementary material, which is available to authorized users.

## Background

Generalized anxiety disorder (GAD) is defined as excessive anxiety and worry about a broad spectrum of events and activities that occurs on most days for at least 6 months [1]. It has been reported that patients with GAD may display abnormal cerebral blood flow [2], however the mechanism is still unclear. Cardio-cerebrovascular modulation, which is related to responses of the autonomic nervous system, has the function of regulating cerebral blood flow [3–5]. Several studies, such as Alkin et al. and Faravelli et al., have focused on cardio-cerebrovascular modulation in panic disorder and have found some significant results [6–8]; however the characteristics of cardio-cerebrovascular modulation in patients with GAD are largely unknown.

Although it has not yet been generally recognized, several studies have reported that GAD is associated with dysregulation of the autonomic nervous system [9, 10]. As an important regulatory factor of cardio-cerebral vascular modulation [3–5], impaired autonomic regulation may influence integrated cardio-cerebrovascular modulation in patients with GAD and affect the cerebral blood flow.

The supine-to-standing test, a transcranial Doppler-based technique, has been developed recently to evaluate the integrated effects of cardio-cerebrovascular modulation [3, 5]. In our previous study, we used the supine-to-standing test and found that cerebrovascular modulation was compromised in patients with anxiety [11]. In the present study, we aimed to explore the characteristics of cardio-cerebrovascular modulation in GAD patients, and to further assess the cardio-cerebrovascular modulation before and after treatment.

## Methods

The prospective study design was approved by the ethics committee of the First Hospital of Jilin University. Written informed consent was obtained from all participants. All methods were performed in accordance with the relevant guidelines and regulations.

### Study design and participants

There are two parts in this study. In part 1, we investigated the characteristics of cardio-cerebrovascular modulation in GAD patients. In part 2, we investigated the changes of cardio-cerebrovascular modulation after treatment in the severe GAD group.

### Part 1

**Fig. 1:**
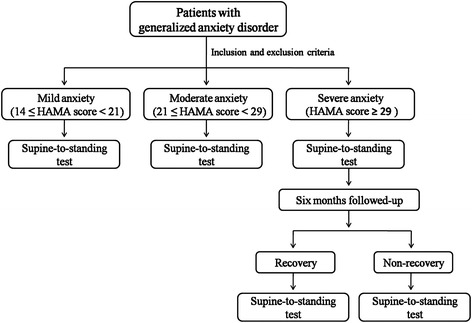
Study design

Male or female patients (aged ≥18 years) who met Diagnostic and Statistical Manual of Mental Disorders, Fourth Edition, Text Revision (DSM-IV-TR) criteria for GAD [12] were enrolled from February 2011 to December 2015 in the outpatient unit, Department of Neurology, the First Hospital of Jilin University. Patients were required to have a Hamilton Rating Scale for Anxiety (HAMA) [13] total score of ≥14, including HAMA items 1 (Anxious Mood) and 2 (Tension) score of ≥2, and a 17-item Hamilton Depression Rating Scale (HAMD) [14] total score of ≤17 at screening and baseline visits. Patients were divided into 3 groups according to the HAMA score: mild anxiety (≥14 and <21), moderate anxiety (≥21 and <29), and severe anxiety groups (≥ 29) (Fig. 1).

Patients were otherwise healthy and the clinical examination consisted of a thorough physical examination, electrocardiography, transcranial Doppler, carotid ultrasound, and cranial computed tomography. Laboratory tests including liver and kidney function tests and hematology profiles were normal. Patients were excluded if they had any concurrent psychiatric disorder other than GAD or prior history of psychiatric disorders. The patients were denied exposure to psychotropic or vasoactive medications for 1 month prior to receiving the supine-to-standing test. Thirty-three age-matched and sex-matched medically and psychiatrically healthy volunteers were recruited as control participants (from healthy participants who were undergoing routine health examinations in our department). Two blinded clinical psychiatrists evaluated the patients’ mental health status. We recorded the dynamic changes of the CBFV and heart rate values in response to an orthostatic challenge using the supine-to-standing test for each participant. Patients received appropriate drugs and psychological treatment provided by a psychiatrist.

### Part 2

We followed up the severe GAD patients for a period of 6 months, and recorded HAMA scores, HAMD scores, and the supine-to-standing test results at the end of follow-up (Fig. 1). The recovery group’s score was HAMA <7 after treatment; the non-recovery group’s score was HAMA ≥14 after treatment.

### Transcranial Doppler protocol

The transcranial Doppler (MultiDop X4, DWL, Germany) protocol was basically in accordance with that described previously [3, 11]. Briefly, signals from the middle cerebral artery CBFV were recorded with a 2-MHz probe through the temporal window at a depth of 50–60 mm. Participants were maintained in a supine position for 3 min, then asked to stand up quickly within 8 s and to remain in the upright position for 2–3 min. After that, the subjects returned to the supine position. The real-time blood flow spectrum was recorded simultaneously. The whole protocol was completed within 15 min [3, 11]. Blood pressure (measured with a manual sphygmomanometer) and heart rate were measured in the supine position and 2 min after standing (or before returning to the supine position).

### Transcranial Doppler curve analysis

For each subject, values of mean CBFV in the supine and upright positions (1–2 min after standing, stable period) were analyzed. The CBFV variation (dynamic changes in the CBFV values) was calculated as the CBFV value in the supine position minus the value in the upright position.

### Statistics

The statistical program for the social sciences 17.0 (SPSS; IBM, West Grove, PA) was used to analyze all the data. One-way ANOVA, Chi-square test, Student’s *t* test and Wilcoxon signed-rank tests were used to compare the values between groups. All the tests were two-tailed, and the level of significance was set at *P* < 0.05.

## Results

### Demographic information

In total, 125 consecutive patients with GAD were included. There were 52 patients with mild anxiety, 43 patients with moderate anxiety, and 30 patients with severe anxiety. Demographic information is shown in Table 1.

**Table 1:** Demographic information

	GAD group (*n* = 125)	GAD group			Healthy group (*n* = 33)
		Mild anxiety	Moderate anxiety	Severe anxiety	
		(*n* = 52)	(*n* = 43)	(*n* = 30)	
Males (%)	39 (31.2%)	21 (40.4%)	10 (23.3%)	8 (26.7%)	8 (24.2%)
Age (years)	39.6 ± 12.2	36.3 ± 12.5	40.4 ± 12.4	44.3 ± 9.5	37.9 ± 16.1
HAMA score	22.0 (18.0-–28.0)	17.5 ± 1.7	24.5 ± 2.3	32.7 ± 2.7	2.9 ± 1.7
CBFV (cm/s)	57.6 ± 11.5	57.4 ± 10.5	57.9 ± 13.0	57.6 ± 11.1	59.5 ± 11.8
Heart rate (beats/min)	71.8 ± 10.4	71.6 ± 9.2	71.3 ± 11.5	72.7 ± 11.0	74.2 ± 10.4
Supine arterial pressure (mmHg)	91.9 ± 6.8	91.0 ± 6.2	93.1 ± 6.1	91.8 ± 8.4	89.5 ± 5.6
Standing arterial pressure (mmHg)	88.4 ± 8.1	86.4 ± 6.7	89.4 ± 7.8	90.4 ± 10.1	86.7 ± 6.9

*GAD* generalized anxiety disorder, *CBFV* cerebral blood flow velocity

In the follow-up of patients with severe anxiety, 1 patient was lost from the study, 1 patient had a HAMA score within 7–13, and 1 patient was diagnosed as GAD associated with depression. In the rest of the 27 patients, 15 patients showed recovery from their symptoms (recovery group, HAMA score, 4.3 ± 1.5), and 12 patients still demonstrated anxiety (non-recovery group, HAMA score, 22.8 ± 6.8).

### Part 1: Characteristics of the parameters in the supine-to-standing test in patients with GAD

#### Variation of CBFV and heart rate in the supine-to-standing test

**Fig. 2:**
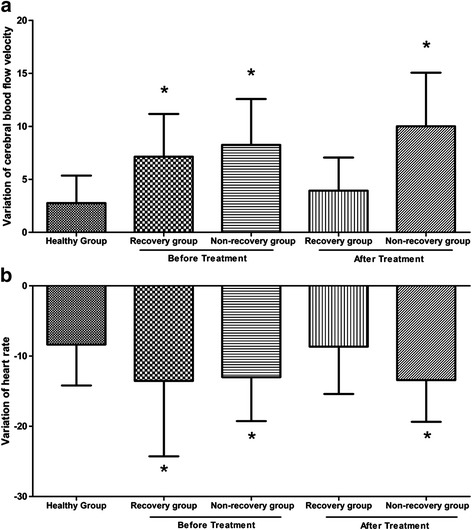
Statistical distributions of variation of cerebral blood flow velocity (CBFV) (**a**), and heart rate (**b**) in each group. The CBFV and heart rate changes in the generalized anxiety disorder (GAD) group, recovery group before treatment, and non-recovery group before and after treatment were significantly higher than in the healthy group

The baseline values of mean CBFV in the GAD group and the healthy group were similar (*t* = 1.9, *P* > 0.05, Additional file 1). However, the CBFV changes from the supine to the upright position differed between the 2 groups. The GAD group displayed more marked CBFV changes than the healthy group (*t* = 8.0, *P* < 0.05, Table 2 and Fig. 2, Additional file 1).

**Table 2:** Variation (Supine - Upright) of CBFV and heart rate in patients with GAD

	Variation of CBFV	Variation of heart rate
GAD group (*n* = 125)	7.34 ± 3.97*	−12.82 ± 10.02*
Mild anxiety (*n* = 52)	7.28 ± 3.82*	−13.56 ± 10.30*
Moderate anxiety (*n* = 43)	7.45 ± 4.18*	−11.52 ± 10.10
Severe anxiety (*n* = 30)	7.31 ± 4.04*	−12.77 ± 9.56*
Healthy group (*n* = 33)	2.75 ± 2.62	−8.36 ± 5.81

*GAD* generalized anxiety disorder, *CBFV* cerebral blood flow velocity

*: *P* < 0.05 for comparing with healthy group

The baseline heart rate in the GAD group and the healthy group were similar (*t* = −1.2, *P* > 0.05). The heart rate changes in the GAD group were significantly increased compared with the healthy group (*t* = −2.7, *P* < 0.05, Table 2 and Fig. 2).

#### Variation of CBFV and heart rate in different anxiety levels

There was no difference in the baseline values of CBFV and heart rate between the mild anxiety, moderate anxiety, and severe anxiety groups (all *P* > 0.05). Similarly, there was no difference in the CBFV and heart rate changes from the supine to the upright position in the 3 groups (all *P* > 0.05, Table 2).

### Part 2: Variation of cardio-cerebrovascular modulation in recovery and non-recovery groups in severe GAD patients

#### Variation of CBFV and heart rate in the recovery group

**Fig. 3:**
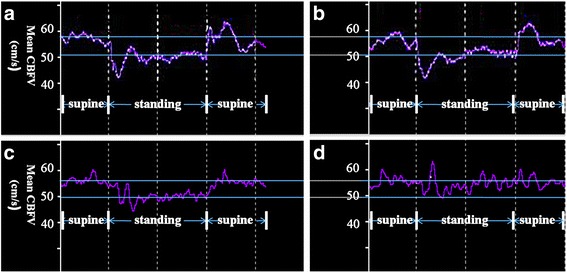
Representative curves of dynamic changes of cerebral blood flow velocity (CBFV) values in the non-recovery (**a** and **b**) and recovery (**c** and **d**) groups. In the recovery group before treatment, the CBFV changes from the supine to upright position were significantly higher than the healthy group (**c**); however, this impairment can be restored to normal after the disappearance of anxiety (**d**). In the non-recovery group, the CBFV changes were significantly higher than the healthy group regardless of treatment (**a** and **b**)

In the recovery group before treatment, the CBFV changes from the supine to the upright position were significantly higher than the healthy group (*t* = 4.5, *P* < 0.05); however, these changes were not observed after the disappearance of anxiety (*t* = 1.4, *P* > 0.05, Table 3, Figs. 2 and 3). The variation tendency of heart rate was similar with CBFV changes (*t* = −2.2, *P* < 0.05, *t* = −0.16, *P* > 0.05; Table 3 and Figs. 2 and 3).

**Table 3:** Variation (Supine - Upright) of CBFV and heart rate in severe GAD before and after treatment

	Variation of CBFV	Variation of heart rate
Recovery group before treatment (*n* = 15)	7.13 ± 4.03*	−13.53 ± 10.74*
Recovery group after treatment (*n* = 15)	3.93 ± 3.13	−8.67 ± 6.73
Non-recovery group before treatment (*n* = 12)	8.22 ± 4.32*	−13.00 ± 6.25*
Non-recovery group after treatment (*n* = 12)	10.03 ± 5.09*	−13.42 ± 5.95*
Healthy group (*n* = 33)	2.75 ± 2.62	−8.36 ± 5.81

*GAD* generalized anxiety disorder, *CBFV* cerebral blood flow velocity

*: *P* < 0.05 for comparing with healthy group

#### Variation of CBFV and heart rate in the non-recovery group

In the non-recovery group, the CBFV changes and heart rate changes from the supine to the upright position were significantly higher than the healthy group regardless of treatment (CBFV changes before treatment, *t* = 5.2, *P* < 0.05; CBFV changes after treatment, *t* = 4.7, *P* < 0.05; heart rate changes before treatment, *t* = −2.3, *P* < 0.05; heart rate changes after treatment, *t* = −2.6, *P* < 0.05; Table 3, Figs. 2 and 3).

## Discussion

In the present study, we found that integrated cardio-cerebrovascular modulation was compromised in patients with GAD. However, this impairment was restored to normal with the disappearance of anxiety.

It has been reported that the lifetime prevalence of GAD is 7.7% in women and 4.6% in men [15]. Patients with GAD often have associated physical symptoms, such as sleep disturbance, gastrointestinal symptoms, and chronic headaches [12, 16]. Recent studies have also suggested that patients with GAD may experience persistent activation of areas of the brain associated with mental activity and introspective thinking following worry-inducing stimuli [16, 17]. Andreescu et al. compared 7 elderly GAD patients and 10 elderly healthy controls using functional MRI. They found that the elderly GAD patients were not effectively engaging the regional cerebral blood flow in the prefrontal cortex in suppressing worry [2]. Zhuang et al. found that patients with GAD have different perfusion characteristics for different anxiety levels [18]. Although several studies demonstrated abnormal cerebral hemodynamics in GAD, the mechanism is still unclear. Cardio-cerebrovascular modulation is one possible mechanism for regulating the cerebral blood flow, and it is a comprehensive reflection of the regulation of autonomic nerves and cerebral autoregulation [3]. Autonomic dysregulation has been reported in patients with GAD [9, 19]. Thus, there is reason to believe that the cardio-cerebrovascular modulation is compromised in patients with GAD. In our previous study, we used the supine-to-standing test and found that cerebrovascular modulation was compromised in patients with anxiety [11]. However, few reports have focused on the characteristics of cardio-cerebrovascular modulation in GAD.

In this study, we used the supine-to-standing test to evaluate the integrated effects of cardio-cerebrovascular modulation in patients with GAD. When a healthy subject stood abruptly, because of the sharp drop in blood pressure, the CBFV curve descended sharply to a lower level, followed by the baroreflex modulation, the CBFV rebounded to the same level as the supine baseline curve or even higher, and then the CBFV was stably maintained at a lower level than the supine baseline, this reflected the final compromised balance of cardiovascular modulation and cerebrovascular modulation. When we analyzed the curve from GAD patients, we found the stable curve 1–2 min after standing was decreased in GAD patients compared with the healthy group. This part of the curve reflects the final compromised balance of cardio-cerebrovascular modulation [3]. In addition, the variation of heart rate reflects autonomic dysregulation, which is an important regulatory factor of cardio-cerebrovascular modulation. It is worth mentioning that there is equal impairment of cardio-cerebrovascular modulation in the mild, moderate, and severe anxiety groups, which reminds us to pay close attention to all kinds of anxiety levels. It is encouraging that the impaired cardio-cerebrovascular modulation can be restored after the treatment. This result is beneficial for patients with GAD, since impaired cardio-cerebrovascular modulation is an important risk factor for stroke [20]. Early diagnosis of impaired cerebrovascular modulation will enable early treatment with intervention therapies in patients with GAD, and thus improve the primary prevention of cerebrovascular disease.

In addition, the diagnosis of GAD mainly relies on scale examination, the accuracy of which is too subjective for further treatment. The supine-to-standing test may become a new objective method to assess the conditions of patients with GAD and can also be used to measure the efficacy of anti-anxiety drugs [21].

This study has some limitations. First, we only followed up patients with severe GAD, making this study insufficiently comprehensive. Second, the high prevalence of GAD in women resulted in a greater number of women than men in this study, which might have led to a sex bias. Third, patients with GAD may present with panic states or sub-threshold panic states, thus, we cannot exclude the effect of panic states on cardio-cerebrovascular modulation. In addition, the small sample size is another limitation of our study.

## Conclusion

Integrated cardio-cerebrovascular modulation was compromised in patients with GAD, and this impairment was restored to normal after the disappearance of anxiety.

## Additional file

Additional file 1: Available data in patients with generalized anxiety disorder and healthy controls. Data of age, supine arterial pressure, standing arterial pressure, cerebral blood flow velocity, Heart rate, and HAMA score in patients with generalized anxiety disorder and healthy controls. (XLSX 14 kb)

## Abbreviations

CBFV: Cerebral blood flow velocity
GAD: Generalized anxiety disorder
HAMA: Hamilton rating scale for anxiety
HAMD: Hamilton depression rating scale
